# Rapid regeneration and ploidy stability of ‘cv IR36’ indica rice (*Oryza Sativa*. L) confers efficient protocol for *in vitro* callus organogenesis and *Agrobacterium tumefaciens* mediated transformation

**DOI:** 10.1186/1999-3110-54-47

**Published:** 2013-10-21

**Authors:** Subramanian Radhesh Krishnan, Arumugam Mohana Priya, Manikandan Ramesh

**Affiliations:** Department of Biotechnology, Alagappa University, Karaikudi, 630 003 Tamil Nadu India

**Keywords:** Transformation, Micropropagation, IR36, ISSR, Ploidy, RAPD

## Abstract

**Background:**

Cereal crops are the major targets for transformation mediated crop improvement and IR36 is an early maturing, high yielding, insect and disease resistant rice variety however, it is abiotic stress sensitive. Hence, development of an efficient and reproducible micropropagation system via somatic embryogenesis and *Agrobacterium tumefaciens* mediated transformation is prerequisite to develop abiotic stress tolerant IR36. Further, Genetic stability of analysis of plantlets through RAPD and ISSR and Ploidy level through Flow cytometry (FCM) measurement of 2C DNA content is necessary for future application of transformed IR36.

**Results:**

In this study, Mature seeds inoculated on (Murashige and Skoog) MS medium with 11.31 μM 2, 4-dichlorophenoxyacetic acid (2, 4-D) and 0.3 μM Kinetin (Kn) had highest callus induction frequency (98%). The highest regeneration frequency (80%) was observed in MS + 13.28 μM Benzyladenine (BA) with 8.06 μM *α*-naphthalene acetic acid (NAA). Randomly Amplified Polymorphic DNA (RAPD), Inter Simple Sequence Repeat (ISSR) and Flow Cytometry (FCM) analysis showed no significant variation in the 2C DNA (0.81 pg/2C) content and Ploidy level between wild type IR36 and *in vitro* maintained rice lines. Of the various OD bacterial culture, an optimum OD of 0.4 and inoculation duration of 10 min resulted in efficient *Agrobacterium*-mediated transformation. β-glucuronidase activity was maximum in callus (99.05%).

**Conclusions:**

These results described here confirm the reliability of this protocol for micropropagation and delivery of desirable gene using *A. tumefaciens* into indica rice.

**Electronic supplementary material:**

The online version of this article (doi:10.1186/1999-3110-54-47) contains supplementary material, which is available to authorized users.

## Background

*Oryza sativa* is one of the popular cereals having high source of dietary carbohydrate and is a staple diet for more than three billion people, supplying 50–80 percent of their daily calorie need (Khush 2005). Hence, there is an urgent need to improve global rice production to meet the demand of ever increasing population. Thus it necessitates manipulation and successful implementation of tissue culture and molecular biological approaches. An efficient plant regeneration system is a prerequisite for genetic transformation of plants and crop improvement (Raemakers et al. 1997 Sanyal et al. 2005 Dabul 2009). Various protocols have been developed to initiate callus from explants such as immature embryos (Seraj et al. 1997 Li et al. 2007 Koetje et al. 1989), mature embryos (Seraj et al. 1997 Ramesh and Gupta 2006 Abe and Futsuhara 1984 Abe and Futsuhara 1986 Wang et al. 1987Azria and Bhalla 2000), root segments (Abe and Futsuhara 1984 Mandal et al. 2003), coleoptile (Oinam and Kothari. 1995 Chand and Sahrawat 1997), and leaf bases (Ramesh et al. 2009). A major hitch in tissue culture is conservation of genetic constancy (Rout et al. 2006). The genetic stability of tissue culture derived plantlets could be analysed through RAPD and ISSR markers (Priya et al. 2011 Shilpha et al. 2013). RAPD is an inexpensive but very sensitive method which requires less amount of genomic DNA (Williams et al. 1990) and ISSR markers are capable to discriminate closely related individuals (Moreno et al. 1998 Fang and Roose 1997). Further the Ploidy level and the 2C DNA content (Swift 1950; Bennett et al. 2000) can be analysed by flow cytometry.

Plant transformation technology has become an adaptable platform for cultivar improvement (Rao et al. 2009). Numerous transformation strategies are available. However, *Agrobacterium*-mediated transformation appears more effective and is the preferred mode because of regenerating plants with low transgene copy number, relatively precise mode of DNA transfer, high efficiency of transformation, transfer of relatively large piece of DNA, less expensive nature (Hiei et al. 1994), more stable over generations and reduced gene silencing associated with integration of TDNAs into euchromatic regions (Barakat et al. 1997 Shou et al. 2004). Though *Agrobacterium*-mediated transformation of several dicotyledonous plants is common, monocots are generally considered recalcitrant to *Agrobacterium* (Mohanty et al. 1999). Furthermore, transformation protocol for generating a large number of transformants is limited to only a few cultivars, indicating that experimental parameters for rice transformation have not been fully optimized (Ozawa 2009).

In order to overcome the major constraint of recalcitrant response of monocot to *in vitro* regeneration and transgene delivery, evaluation of regenerable explants material is becoming vital. Transient GUS expression was carried out to screen the explant source. Though stable transformation is necessary for gene integration analysis, it is time consuming (Yasmin and Debener 2010) but transient GUS expression is more cost effective (Wroblewski et al. 2005). In the present investigation a fast and reproducible protocol for an effective embryogenic callus induction, regeneration and *Agrobacterium*-mediated transformation of IR36, an extremely drought sensitive cultivar (Atlin et al. 2012) has been reported and this could be further exploited to raise transgenic indica rice.

## Methods

### Plant material

IR36 seeds were procured from Tamil Nadu Rice Research Station, Aduthurai, Tamil Nadu, India. It was ensured that the seeds were healthy and disease free and then manually dehusked and surface sterilized with 70% (v/w) ethanol for 45 seconds followed by 0.1% mercuric chloride for 3 minutes and then thoroughly washed thrice with autoclaved distilled water, aseptically in laminar air flow chamber.

### Callus induction from different explants

Surface sterilized seeds were transferred to sterile tissue papers to remove the moisture, using sterile forceps. These seeds were inoculated on autoclaved MS medium (Murashige and Skoog. 1962) supplemented with 2,4-D (2.26, 4.52, 6.78, 9.04, 11.31, 13.57, 15.83, 18.09 μM) and combination of 2,4-D (11.31 μM) with Kn (0.02, 0.20, 0.30, 0.40, 0.50, 0.60, 0.70, 0.80, 0.90, 0.22 μM) and with BAP (0.44, 0.88, 1.32, 1.76, 2.20, 2.64, 3.08, 3.52, 3.96, 4.40 μM) separately. The pH of media was adjusted to 5.8 before autoclaving it for 15 min at 121°C. The cultures were incubated in the dark at 24 ± 2°C for four weeks for callus induction. The creamy white, friable and nodular embryogenic calli were excised and transferred to the same fresh medium for further growth and embryo maturation.

In addition to seeds, intact explants were used as plant material for micropropagation experiments and transformation, to obtain these explants (leaf base, coleoptile, leaf blade and root) seeds were initially germinated on half-strength MS media for a period of 1 week. Then each explant was transferred to MS with 11.31 μM 2, 4-D and 0.3 μM Kn for callus induction. A total of 16 explants were used and the experiment was done in triplicates.

### Regeneration from callus

Approximately 5 mm (diameter) of the subcultured calli were exposed to two treatments, MS medium supplemented with various concentrations of BAP (0.44, 0.88, 1.32, 1.76, 2.20, 2.64, 3.08, 3.52, 3.96, 4.40, 6.65, 8.86, 11.07, 13.28, 15.49, 17.70 μM), then with combination of 13.28 μM BAP and NAA (0.53, 1.07, 1.61, 2.1, 2.6, 3.22, 3.76, 4.3, 4.83, 5.37, 8.06, 10.7, 13.34, 15.98, 18.62, 21.26). Hundred explants were used in each concentration and were done in triplicates to get the significant regeneration efficiency. The cultures were maintained *in vitro* at 24°C under long-day light conditions with a 16/8 h day/night photoperiod, with an average irradiance of 50 mmol m^-2^ s^-1^.

### Rooting and acclimatization

Regenerated shoots (around 8 cm after 21 days in regeneration medium) were aseptically excised and transferred to half strength MS medium with 30 g L^-1^ sucrose. The cultures were kept for a photoperiod of two weeks. After the complete initiation of roots, the agar attached on roots were removed and were washed with running tap water and then transferred to a tray with vermi compost and peat soil (1:1 ratio) for a period of two weeks. Then finally transferred to green house condition and watered daily.

### Genetic stability analysis

#### RAPD and ISSR markers

Analysis of the genetic stability was carried using RAPD and ISSR (SIGMA, USA) markers. The plant genomic DNA of germinated, *in vitro* regenerated, *in vitro* germinated and hardened plantlets was extracted using HiPurA kit (HiMEDIA, India) and its quality was checked by resolving in 0.8% agarose gel. DNA concentration and purity was measured using spectrophotometer (Hitachi U2800) at 260 nm. 50 ng of total genomic DNA was used as template for a 25 μl reaction mixture consisting of 2.5 μl of 10× buffer, 2 mM MgCl_2_, 200 μM of dNTPs, 5 μM primer, 1 U Taq DNA polymerase (Thermo Scientific). The PCR conditions were as follows, initial denaturation at 94°C for 7 min, followed by 40 cycles denaturation at 94°C for 1 min, annealing at 37°C for 1 min and extension for 2 min at 72°C, followed by a final extension for 7 min at 72°C, using a programmable thermal cycler (Eppendorf).

### Flow cytometry analysis

Nuclear suspension was prepared using Two Step protocol (Dolez. 2007, Otto. 1990). 20 mg of young leaves of rice plantlet were placed at the centre of petri plate and to this, added 2 ml of ice-cold Otto I solution [0.1 M Citric acid, 0.5% (vol/vol) Tween 20]. Using a sterile scalpel the leaves were chopped into small pieces and the homogenate was mixed by pipetting slowly (without air bubbles), and is filtered through a 42-nm nylon mesh. Then pelleted the nuclei at 150 g/5 min. Slowly the supernatant was removed and to the pellet added 100 μl fresh ice-cold Otto I solution. Then to this 1 ml of Otto II solution [0.4 M Na2HPO4.12H_2_O] was added. Finally Propidium Iodide (PI-50 mg/ml) was added and shaken gently and simultaneously treated with RNAse.

PI stained nuclei were analysed through flow cytometer (FACS Aria III (BD Biosciences). Batch analysis report of each sample was calculated using FACSDiva Version 6.1.3 software. IR36 wild type (Tamil Nadu Rice Research Station, Aduthurai) with 2C DNA value 0.90 pg (Martinez et al. 1994) was used as external standard. The 2C DNA content of randomly chosen three samples from each of germinated, *in vitro* germinated, *in vitro* regenerated and hardened leaves tissues were calculated as according to (Dolezel et al. 2007). The 2C genome sizes were obtained by the conversion (1 pg DNA to 978 Mbp) given by (Dolezel et al. 2003). DNA fluorescence index (Alan et al. 2007) shows how close are the control and the samples tested in its 2C DNA content.

### Bacterial strain and vector

EHA105 containing the disarmed *Ti* plasmid and the binary vector pCAMBIA1301 containing the *GUS* gene with castor bean catalase intron, the kanamycin phosphotransferase II (*ntpII*) gene and hygromycin phosphotransferase (*hpt*) gene, conferring resistance to kanamycin and hygromycin respectively.

### *Agrobacterium-* mediated transformation

Overnight culture of *Agrobacterium tumefaciens* was adjusted to various OD (0.2 - 1.0) by suspending the pellet in 30% liquid MS medium. About 5 mm diameter of sub cultured embryogenic calli were used for infection and were co-cultivated for different time periods (5–25 min) with different OD cultures. The calli were blot dried with sterile tissue paper. These calli were transferred to co-cultivation medium incorporated without and with various concentrations of AS (100–500 μM) with Whatmann #1 filter paper on the surface. The infected calli were kept under incubation (24 ± 2°C) for 48 to 72 hours in dark.

### GUS assay of the putative transformants

After 48 to 72 hrs of co-cultivation with *Agrobacterium*, the infected explants embryogenic calli, coleoptiles, leaf bases, leaf blades and root tips were subjected to GUS assay. For histochemical detection, GUS assay buffer having 50 mM phosphate buffer, TritonX-100, 20% methanol, and 1 mM substrate 5-bromo, 4-chloro, 3-indolyl β-D-glucuronide (Jefferson. 1987) was used. The samples were then incubated in dark at 37°C for 24 to 48 hrs. The transformation frequency was observed by calculating the ratio between numbers of calli expressing GUS with the total number of calli (25 explants per experiment, in triplicates) kept for assay. Additionally the transient GUS expression in explants were confirmed by taking cross sections of these GUS-positive explants and were observed under bright field microscope (Nikon H550L, Nikon, Tokyo, Japan).

### Statistical analysis

Data were recorded for per cent callus induction, shoot regeneration, GUS expression and ploidy analysis. All the experiments were repeated thrice and done in a completely randomized design. Data were subjected to one way analysis of variance (ANOVA). Values at *P*<*0.5* level of significance were used for mean comparison.

## Results

### Effect of 2,4-D, Kn and BAP on callus induction

In order to evaluate the effect of growth hormones on callus induction different concentrations of 2,4-D alone was incorporated in different media – MS, B5 (Gamborg et al. 1968) and N6 (Chu 1978). MS medium with 11.31 μM 2,4-D gave good callus induction with 140 mg fresh weight. Comparatively the callus was friable, embryogenic and creamy white in texture than those from B5 and N6 medium that produced brown and slimy callus. Combination of 2, 4-D (11.31 μM) with various concentrations of BAP and Kn incorporated in MS were used for callus induction. Embryogenic scutellum derived calli formation from seed explants was favoured in the presence of 2,4-D and 2,4-D with BAP whereas 2,4-D with Kn had resulted in maximum callus induction of 98% which is significantly higher than the maximum callus induction at 11.31 μM 2,4-D (93.75%) alone or in combination with 0.44 μM BAP (85%) (Table 1). Furthermore, increase in callus induction was also accompanied by marked increase in calli fresh weight of 161 mg. A comparison of the maximum fresh weight of callus obtained using 2,4-D (15.83 μM) and 2,4-D (11.31 μM) with BAP (0.44 μM) showed decline in callus fresh weight 145 mg and 154 mg respectively.

**Table 1:** **Effect of plant growth regulators (PGRs) on callus induction from mature seed after 28 days of incubation**

Concentration of 2,4-D (μM)	Concentration of Kn (μM)	Concentration of BAP (μM)	Average callus induction (%)	Average mean F.Wt (mg)
Control (MS alone)	-	-	6.25 ± 0	41.3 ± 2.1
11.31	-	-	93.75 ± 6.25*	140.24 ± 0.9*
11.31	0.02	-	63.17 ± 0.76	154.73 ± 2.44
11.31	0.20	-	87.00 ± 0.50	148.70 ± 5.28
11.31	0.30	-	98.00 ± 0.87*	161.28 ± 1.28*
11.31	0.40	-	62.00 ± 0.50	157.03 ± 4.87
11.31	0.50	-	55.42 ± 0.88	150.05 ± 5.93
11.31	0.60	-	44.58 ± 0.88*	151.29 ± 8.52
11.31	0.70	-	36.67 ± 0.76*	146.31 ± 3.61
11.31	0.80	-	98.17 ± 0.29	146.56 ± 3.78
11.31	0.90	-	43.08 ± 0.95*	150.46 ± 7.37
11.31	0.22	-	11.67 ± 0.76*	142.83 ± 1.06
11.31	-	0.44	85.00 ± 3.50	154.09 ± 4.67
11.31	-	0.88	73.16 ± 2.02	147.13 ± 6.37
11.31	-	1.32	76.08 ± 2.79	148.87 ± 0.89*
11.31	-	1.76	52.16 ± 2.02*	135.43 ± 10.79
11.31	-	2.20	50.66 ± 3.06*	146.52 ± 8.93
11.31	-	2.64	58.83 ± 3.33*	144.79 ± 4.21
11.31	-	3.08	79.66 ± 1.53	150.50 ± 8.08
11.31	-	3.52	75.83 ± 1.04	140.67 ± 0.50
11.31	-	3.96	68.75 ± 0.00	142.65 ± 12.23
11.31	-	4.40	61.5 ± 1.32	128.50 ± 0.47*

-Nil.

Values represent means ± standard deviation; * indicates significance at the level of *P < 0.5.*

### Influence of different explants on callus induction

**Figure 1:**
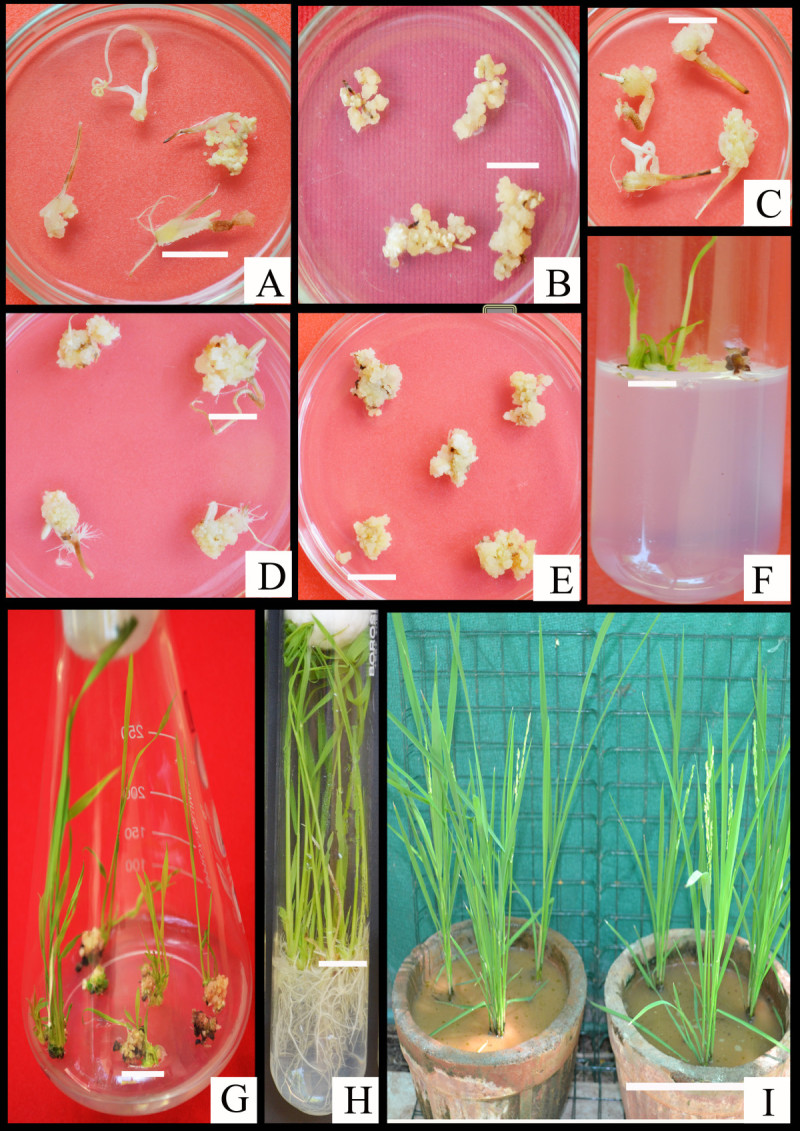
**Stages of** ***in vitro*** **propagation and plantlet establishment.** Calli derived from **A)** Coleoptile (bar = 13 mm), **B)** Root (bar = 10 mm), **C)** Leaf base (bar = 8 mm), **D)** Mature seed derived calli (1 month old) (bar = 11 mm), **E)** Embryogenic calli after subculture (arrows indicate somatic embryos) (bar = 7.5 mm), **F)** Shoot primordia from embryogenic clumps on regeneration medium (bar = 12.5 mm), **G)** Multiple shoot formation and proliferation after 21 day (bar = 22 mm), **H)** Shoots rooted on half-strength MS (bar = 12.5 mm), **I)** 4-months old greenhouse established plants, bar = 95 mm.

**Figure 2:**
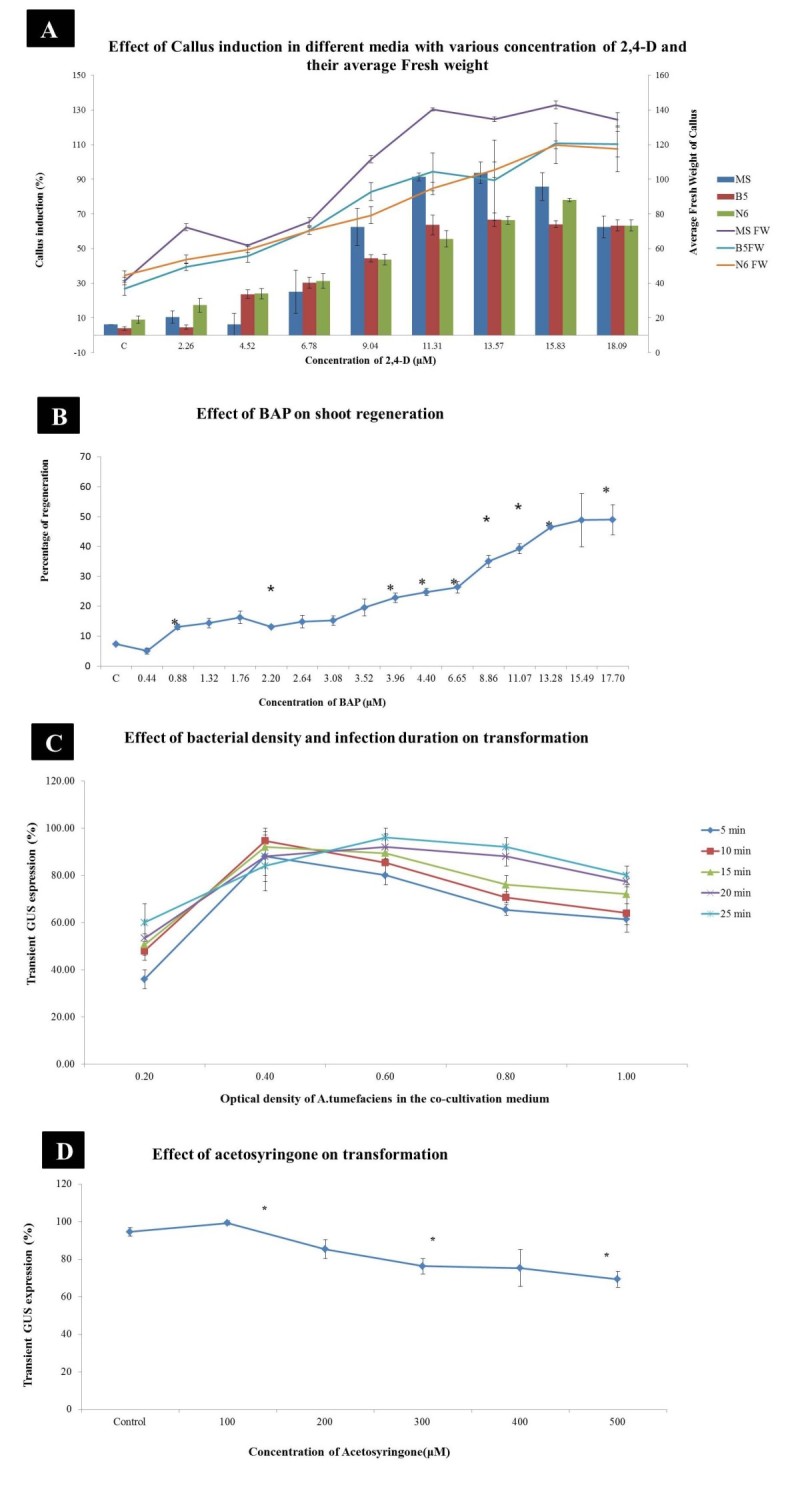
**Effect of Different Growth Hormones and Bacterial density in Tissue Culture and Transformation. A)** Effect of 2,4-D on callus induction, **B)** Effect of BAP on shoot regeneration, **C)** Effect of bacterial density and infection duration on transformation, **D)** Effect of acetosyringone on transformation.

All tested explants showed callus induction as well as callus maturation on MS supplemented with 11.31 μM 2,4-D and 0.30 μM Kn (Figures 1A, B, C, D, and 2A). Among these the mean average of callus induction was 53.6%, 47.3%, 99%, for coleoptile, leaf base and mature seed (Table 2). The results indicate lowest callus induction of 39.6% from root and the absence of callus induction from leaf blade. Subculture allows callus to gain mass in addition to increase in somatic embryos (Figure 1E).

**Table 2:** **Callus induction and histochemical GUS expression of various explants of IR36**

Explant	Average CI (%)	Average F.Wt (mg)	Transformation efficiency (TE %)
Callus	-	-	99.05 ± 1.65
Coleoptile	53.66 ± 6.65	95.60 ± 3.17	87.61 ± 9.18
Leaf base	47.33 ± 1.52	83.46 ± 2.20	85.71 ± 7.55
Mature seed	99.00 ± 0.87	160.76 ± 0.87	50.47 ± 0.00
Leaf blade	0	-	49.52 ± 10.03
Root	39.66 ± 1.15	31.66 ± 3.36	58.09 ± 9.18

-Not applicable.

TE is number of putative transformants/total number of explants per treatment.

### Regeneration

For further regeneration experiments scutellar derived calli were used. After 7 days of culture on various combinations of hormones, green shoot primordia appeared, however more shoot buds were observed in calli on BAP with NAA (Figure 1F). According to the results of the present study, 13.28 μM BAP and 8.06 μM NAA showed 80% regeneration (Table 3) with average shoot height of 17.22 cm (Figures 1G, and 2B). On the other hand evaluation of shoot regeneration as well as shoot height in both treatments with BAP alone or BAP with different combinations of NAA, shown to have reduction in regeneration and mean shoot height after further increase in hormone concentration.

**Table 3:** **Effect of plant growth regulators (PGRs) on regeneration from seed derived calli after 21 days of inoculation**

Concentration of BAP (μM)	Concentration of NAA (μM)	Average regeneration (%)	Average mean of shoot height (cm)
13.28	-	66.0 ± 1.0	7.39 ± 0.43
13.28	0.53	38.2 ± 0.2*	8.73 ± 0.36
13.28	1.07	41.0 ± 2.0*	8.39 ± 0.25
13.28	1.61	49.33 ± 1.5*	9.22 ± 0.19
13.28	2.1	54.60 ± 0*	9.33 ± 0.03
13.28	2.6	57.2 ± 8.5*	8.84 ± 1.20
13.28	3.22	61.133 ± 1.02	14.59 ± 2.35
13.28	3.76	51.86 ± 5.7	13.39 ± 0.37*
13.28	4.3	62.0 ± 2.0	11.63 ± 0.37*
13.28	4.83	65.73 ± 1.2	13.07 ± 0.65*
13.28	5.37	65.0 ± 4.0	14.19 ± 1.64
13.28	8.06	80.00 ± 0.2*	17.22 ± 0.99*
13.28	10.7	75.06 ± 3.5*	17.09 ± 0.50*
13.28	13.34	76.80 ± 0*	15.28 ± 1.0*
13.28	15.98	64.46 ± 0.5	16.06 ± 0.87*
13.28	18.62	56.40 ± 0*	14.91 ± 0.70*
13.28	21.26	52.0 ± 10*	12.55 ± 0.67*

-Not applicable.

Values represent means ± standard deviation; * indicates significance at the level of *P < 0.5.*

### Rooting and acclimatization

Finally elongated shoots were rooted on half-strength MS media. Rooting of green, healthy regenerated shoots on half-strength MS also promoted development of multiple (2–3) secondary shoots (Figure 1H). Properly developed roots appeared after 2 weeks. The plantlets subjected to green house condition also appeared healthy without any morphological changes. Rooting and acclimatization of plantlets resulted in multiple tillers with average height of 82 cm without any deleterious effects on their survival (Figure 1I).

### Effect of bacterial density on transformation

The influence of bacterial density on transformation is significant as the early stage after *Agrobacterium* infection should not be hampered by bacterial overgrowth. Hence, in order to determine the effect of bacterial density on transient GUS expression, calli infected with various concentration of *Agrobacterium* OD_600_ (0.2, 0.4, 0.6, 0.8, 1.0) were monitored. The significant difference was observed between 0.4 and 1.0 OD infected calli marked by high infection rate (Figure 2C). On the other hand significant difference was not observed among other OD whereas tissue necrosis was seen. The optimum OD was 0.4 manifested by significant GUS expression.

### Effect of infiltration and co-cultivation duration

To determine the influence of *Agrobacterium* infection duration on transient GUS expression, various time intervals (5, 10, 15, 20, 25 min) were chosen. Exposure of the explants to 0.4 OD *Agrobacterium* for 10 min was found to be optimum as significant GUS staining was observed whereas 15, 20 and 25 min of infiltration besides resulting in GUS signals lead to contamination (Figure 2C). Co-cultivation for 2 days showed better infection without browning or *Agrobacterium* overgrowth (Data not shown).

### Effect of acetosyringone

**Figure 3:**
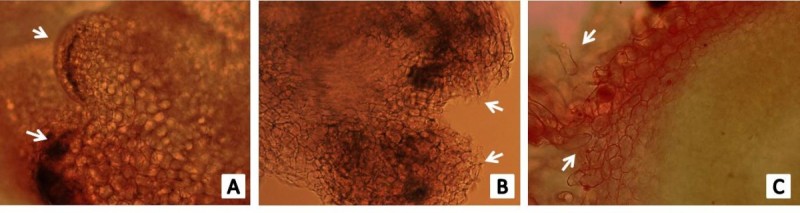
**Development of somatic embryos.** Cross section of **A)** one month old callus with somatic embryo (Arrows indicate globular regions), **B)** Post globular embryo and **C)** Post globular germinating regions of IR36 rice.

Further investigation was carried out to determine the optimum concentration of acetosyringone during co-cultivation, for which various concentration (100, 200, 300, 400, 500 μM) of AS were incorporated separately. Our analysis revealed difference in GUS expression between infection in the presence and absence of AS. However, significant level of GUS expression was observed at 100 μM AS after which GUS expression combined with necrosis was observed (Figure 2D). Cross sections of callus were taken to study the development of somatic embryogenesis. Seed derived callus showed globular embryos after one month of inoculation (Figure 3).

### Histochemical GUS expression analysis

**Figure 4:**
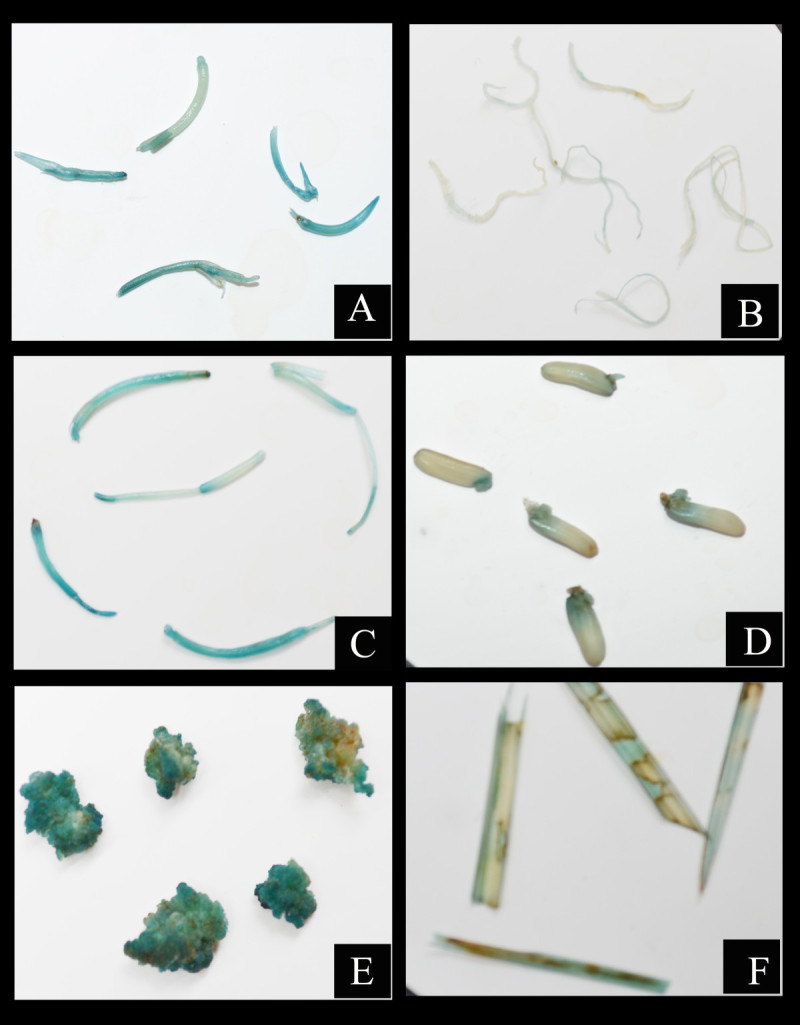
**Histochemical GUS activity in different tissues of** ***A.tumefaciens*** **infected IR36. A)**, **C)** Dark blue staining in coleoptile and leaf base, **D)** **E)** Strongly stained embryogenic calli and mature seed **B)**, **F)** slightly stained root and leaf blade.

**Figure 5:**
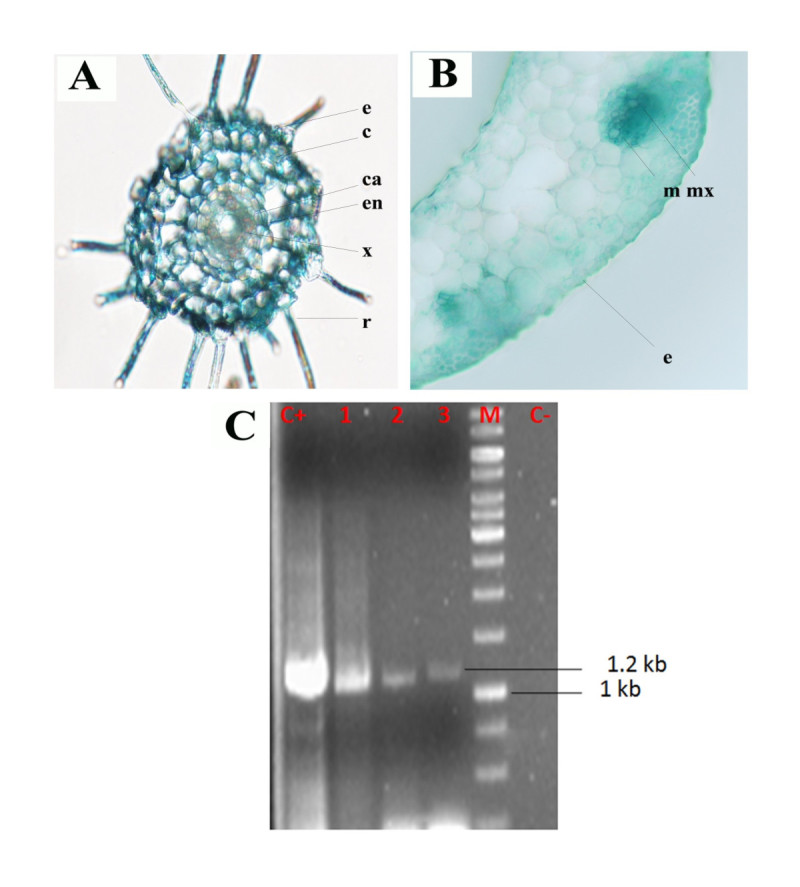
**Cross section examination and PCR analysis of transformants A) Radial root tissue sections of Root (e-epidermis, c-cortex, ca-cambium, en-endodermis, x-xylem, r-root hair), B) Leaf base (m – mesophyll, mx – metaxylem, e – epidermis), C) PCR of GUS positive samples (Lane C + pCAMBIA plasmid, Lane: 1–3 GUS positive samples, Lane M: 1Kb ladder and Lane C- untransformed sample).**

Selection of suitable explants is vital for efficient transformation. Hence the *Agrobacterium* infected explants were screened using GUS assay. Transformation efficiency is expressed as ratio of number of GUS-positive calli to initial number of explants inoculated. Generally all tissue types responded positively to histochemical assay (Figure 4), blue stain was observed in all the explants but stronger in calli (99%) followed by coleoptiles (87.6%), leaf bases (85.7%), root (58%), mature seed (50.47%) and leaf blade (49.5%) (Table 3). Furthermore, cross section of GUS-positive leaf bases were observed under bright field microscope. The arrows designate the region where GUS expression was maximum in the cross section. In all the explants observed the GUS staining was limited to vascular cells and was high in the regions of metaxylem (Figure 5).

### Assessment of genetic stability by molecular markers

**Figure 6:**
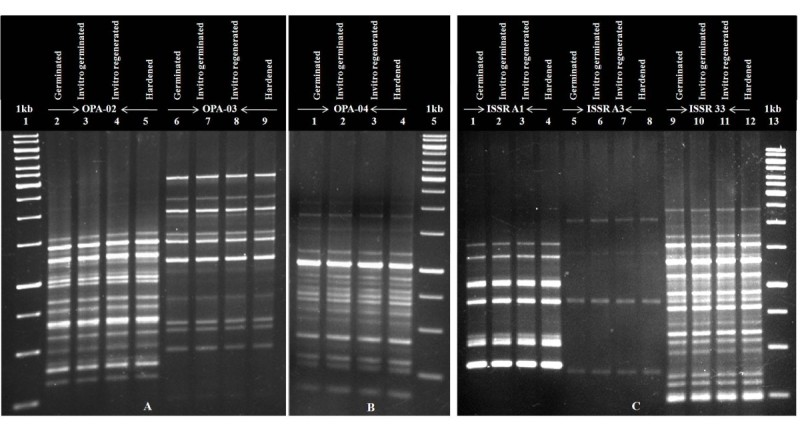
**RAPD and ISSR profile analysis A) Primers OPA-02, OPA-03;** **B) OPA-04;** **C) ISSR A1, ISSR A3, and ISSR 33.**

Ten RAPD and five ISSR primers were used to study the genetic stability of germinated, *in vitro* germinated, *in vitro* regenerated and hardened plantlets (Additional file 1). A total of 158 bands were obtained and no polymorphic band pattern was observed. RAPD and ISSR primers produced amplicons with a range of 250–4000 bps (Figure 6). This ensures that there is no genetic variation among the wild type and tissue culture derived plantlets.

### Ploidy level and 2C DNA content analysis

Flow cytometry analysis of tissue culture derived samples with wild type (IR36) nuclei as external standard provided average mean 2C DNA content of 0.81 pg/2C which was not that significantly different as reported by Martinez et al. 1994 (0.90 pg/2C in IR36). The Propidium Iodine-Absorbance (PI-A) peaks produced by the samples gave an average mean Ploidy level of 24522.58 (Additional file 2). Further the DNA Fluorescence index for wild type and *in vitro* derived samples was found to be similar (Table 4).

**Table 4:** **Nuclear DNA content assessment between wild type and tissue culture derived IR36 samples**

Material donor*	Number of days old	DNA fluorescence index^1^	Average mean ploidy^2^	Average nuclear DNA content^3^(pg/2c)	Average 2c genome size^4^ (Mbps)
Germinated	40	0.942	25798.33	0.85	829.38
In vitro Germinated	45	0.881	24114.00	0.79	775.23
In vitro Regenerated	45	0.873	23901.00	0.79	768.39
Hardened	40	0.887	24277.00	0.80	780.47

^*^Sample Size: Three samples each.

^1^DNA fluorescence index (DI = 2CIR36 sample/2CIR36 wild type) (Alan et al. 2007).

^2^Average PI-A mean value of P2 population.

^3.^ $\mathrm{SamplePloidy}\left(\mathrm{integer}\right)=\mathrm{ReferencePloidy}\times \frac{\mathrm{MeanpositionoftheG}1\mathrm{samplepeak}}{\mathrm{MeanpositionoftheG}1\mathrm{referencepeak}}$

^4^DNA content 1 pg DNA = 978 Mbp (Dolezel et al. 2003.

## Discussion

The aim of this research was to establish efficient micropropagation of IR36 using optimum hormones for somatic embryogenesis and regeneration as well as efficient transformation from various explants. Though various reports are available for micropropagation and transformation of *Oryza sativa* the analysis using different explants source together with the assessment of each explant for transformation is needed. And the present study reports this, in addition to this the genetic stability analysis through three different approaches assure the reliability and applicability of this protocol for other *Oryza sativa* rice varieties. Somatic embryos play a more significant role in micropropagation than any mode of organogenesis and are vital tool for plant transformation either by *Agrobacterium* or biolistic methods (Alaiwi et al. 2012). *Agrobacterium tumefaciens* strain EHA105 harboring a binary vector pCABIA1301 carrying the *UidA* reporter gene and hygromycin phosphotransferase gene under a constitutive CaMV35S promoter was used. Mature seed giving high frequency callus induction (98%) and regeneration (80%) is considered to be the key explant material for *in vitro* propagation and further transformation. This high efficiency reflects the fact that this is widely used for *in vitro* propagation. Wani (Wani et al. 2011) reported the efficiency of scutellar derived calli over other explants derived calli for regeneration. Proliferation of calli was achieved on transfer of the one month old calli to the same fresh medium this in line with the previous report. Breaking of one month old calli resulted in browning consequent to phenolic production and leads to poor callus proliferation and somatic embryogenesis as well as two-month-old hard friable calli were able to withstand *Agrobacterium* infection efficiently (Kumar et al. 2005). On the contrary it has been reported that the regeneration potential of the callus was greatly influenced by the callus age and younger the callus the higher the regeneration potential (Jain AK and Datta RK 1992 Sahoo et al. 1997). The maximum percentage of regeneration using BAP alone was 66%. However, combination of the same concentration of BAP (11.07 μM) with NAA 8.06 μM resulted in 80% of regeneration. Similar finding has been reported already, stating that the interaction of auxin and cytokinin is important for *in vitro* organogenesis (Fan et al. 2011). The mean average of callus induction varied with the type of explants. The maximum callus induction of 98% was observed from mature seed. Variation in callus induction frequency was observed with different explants, coleoptile (53.6%), leaf base (47.3%), root (39.6%). whereas callus failed to induce from leaf explant this is in concordance with previous report from Poaceae family. Friable callus from leaf explants was unsuccessful because they may not be a good source for callus induction (Valentine et al. 2010). The RAPD and ISSR profiling of wild type and tissue culture derived plantlets showed no polymorphism in band patterns. This ensures that there is genetic veracity among the germinated, *in vitro* germinated, *in vitro* regenerated and hardened plantlets. The flow cytometry analysis of the *in vitro* regenerated IR36 and the wild type showed no variations in their Ploidy levels.

Among the several factors taken into consideration the concentration of *Agrobacterium* inoculum in the infection medium is an important factor to develop highly efficient transformation protocol (Li et al. 2007; Gao et al. 2009). Decrease in transformation frequency with increase in bacterial intensity was observed. Our result is in accord with the previous report of poor GUS signal at higher densities (Yasmin and debener 2010) and explant tissues were almost wholly colonized by the bacteria, elimination of which also becomes more difficult (Jha et al. 2011). In monocots, incorporation of phenolic compound acetosyringone during co-cultivation supports the gene transfer (Stachel et al. 1985). Presence of acetosyringone (AS) was found to be essential for transformation (Mohanty et al. 1999) whereas no transformation occurred in the absence of acetosyringone (Hoque et al. 2005). Among the various concentrations tested 100 μM AS resulted in significant GUS expression. Co-cultivation in the presence of 100 μM acetosyringone was found to be the most suitable for optimum transformation (Kumar et al. 2005; Svabova and Griga 2008; Karthikeyan et al. 2012). Transient GUS expression was observed almost in all explants due to the promoter used but with variation in percent response and staining. The CaMV 35S promoter is expressed in all plant organs (Odell et al. 1985). The importance of the starting material for transformation and performance of GUS assay in the tissues immediately after infection as an indication for preferable tissue has been reported (Hiei et al. 1994). The frequency of transformation was high in all the explants except root and leaf blade.

It is evident that the high frequency somatic embryogenesis mediated by callus of various explant source and efficient regeneration demonstrated here also augments frequency of transformation. These results insist that now it is possible to introduce desirable gene efficiently into indica rice cultivar IR36 through *A. tumefaciens*.

## Conclusions

The current study besides describing optimization of hormones for invitro propagation and conditions for *Agrobacterium*-mediated transformation, highlights the genetic stability which is mandatory for tissue culture derived and transformed plantlets. To date, for the first time a highly reproducible protocol has been standardized for efficient in vitro propagation of IR36. High frequency embryogenesis mediated by callus of various explants source and efficient regeneration also augments frequency of transformation. The results insist that now it is possible to introduce desirable gene efficiently into indica rice cultivar IR36 through *Agrobacterium* tumefaciens. The Ploidy level of regenerated and wild type and their genetic stability has been confirmed by FCM and RAPD, ISSR markers respectively.

## Electronic supplementary material

Additional file 1: Details of Primers used for RAPD and ISSR based genetic variances. (DOC 36 KB)

Additional file 2: **Flow cytometer histograms of IR36 A) Germinated, B) Invitro Germinated, C) Invitro Regenerated and D) Hardened.** [X axis- Nucleus Count, Y axis- Propidium Iodide Absorbance (PI-A)]. (JPEG 100 KB)

Below are the links to the authors’ original submitted files for images.

Authors’ original file for figure 1

Authors’ original file for figure 2

Authors’ original file for figure 3

Authors’ original file for figure 4

Authors’ original file for figure 5

Authors’ original file for figure 6

Authors’ original file for figure 7

## Abbreviations

AS: Acetosyringone
BA: Benzyladenine
CaMV 35S: Cauliflower mosaic virus promoter
FCM: Flow cytometry
GUS: *β*–Glucuronidase
Hpt: Hygromycin phosphotransferase
Kn: Kinetin
MS: Murashige and Skoog medium
NAA: *α*-naphthalene acetic acid
2: 4-D: 2,4-Dichlorophenoxy acetic acid.
