# Supplementary material for: Rapid regeneration and ploidy stability of ‘cv IR36’ indica rice (Oryza Sativa. L) confers efficient protocol for in vitro callus organogenesis and Agrobacterium tumefaciens mediated transformation
Source: Bot Stud. 2013 Oct 21;54:47. doi: 10.1186/1999-3110-54-47 (PMC5430341; doi:10.1186/1999-3110-54-47)
Supplement: Supplementary file 1 — Additional file 1: Details of Primers used for RAPD and ISSR based genetic variances. (DOC 36 KB) [file 40529_2013_97_MOESM1_ESM.doc]

**Additional table. Details of Primers used for RAPD and ISSR based genetic variances**

| **S. No** | **Primers** | **Primer sequence (5´–3´)** |
| --- | --- | --- |
| 1 | OPA-02 | GTGAGGCGTC |
| 2 | OPA-03 | AGTCAGCCAC |
| 3 | OPA-04 | AATCGGGCTG |
| 4 | OPB-04 | GGACTGGAGT |
| 5 | OPB-05 | TGCGCCCTTC |
| 6 | OPC-02 | GTGAGGCGTC |
| 7 | OPC-07 | GTCCCGACGA |
| 8 | OPC-16 | CACACTCCAG |
| 9 | OPD-11 | AGCGCCATTG |
| 10 | OPD-18 | GAGAGCCAAC |
| 11 | ISSR A1 | GAGAGAGAGAGAGAGA |
| 12 | ISSR A3 | ACACACACACACACACC |
| 13 | ISSR 33 | AGAGAGAGAGAGAGAGA |
| 14 | ISSR 35 | AGAGAGAGAGAGAGAGT |
| 15 | ISSR 67 | TCTCTCTCTCTCTCCC |

S.No. 1 to 10 RAPD primers; S.no. 11 to 15 ISSR primers
