# Supplementary figures and images for: Rapid regeneration and ploidy stability of ‘cv IR36’ indica rice (Oryza Sativa. L) confers efficient protocol for in vitro callus organogenesis and Agrobacterium tumefaciens mediated transformation
Source: Bot Stud. 2013 Oct 21;54:47. doi: 10.1186/1999-3110-54-47 (PMC5430341; doi:10.1186/1999-3110-54-47)

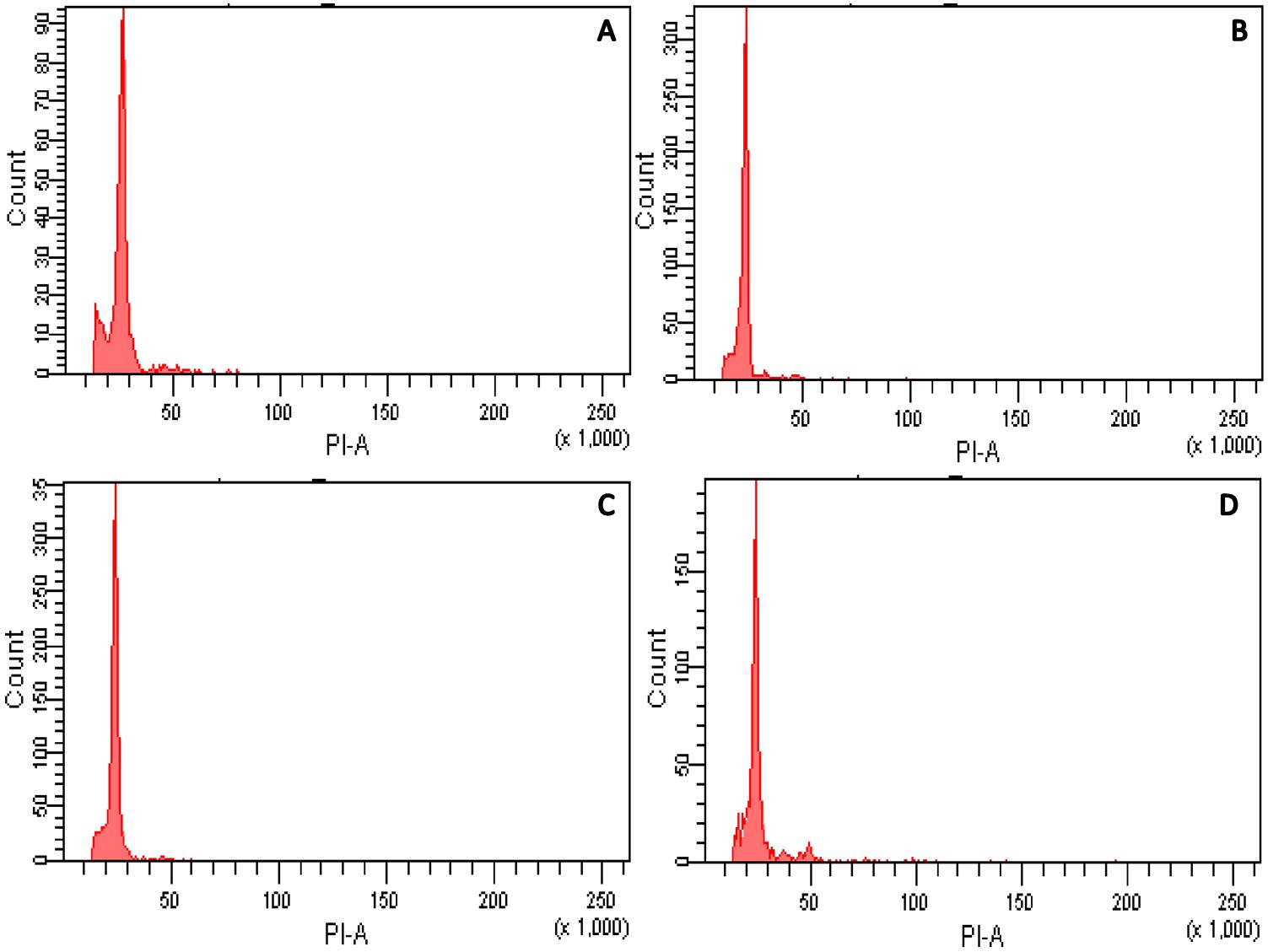

Supplement: Supplementary file 2 — Additional file 2: Flow cytometer histograms of IR36 A) Germinated, B) Invitro Germinated, C) Invitro Regenerated and D) Hardened. [X axis- Nucleus Count, Y axis- Propidium Iodide Absorbance (PI-A)]. (JPEG 100 KB) [file 40529_2013_97_MOESM2_ESM.jpeg]

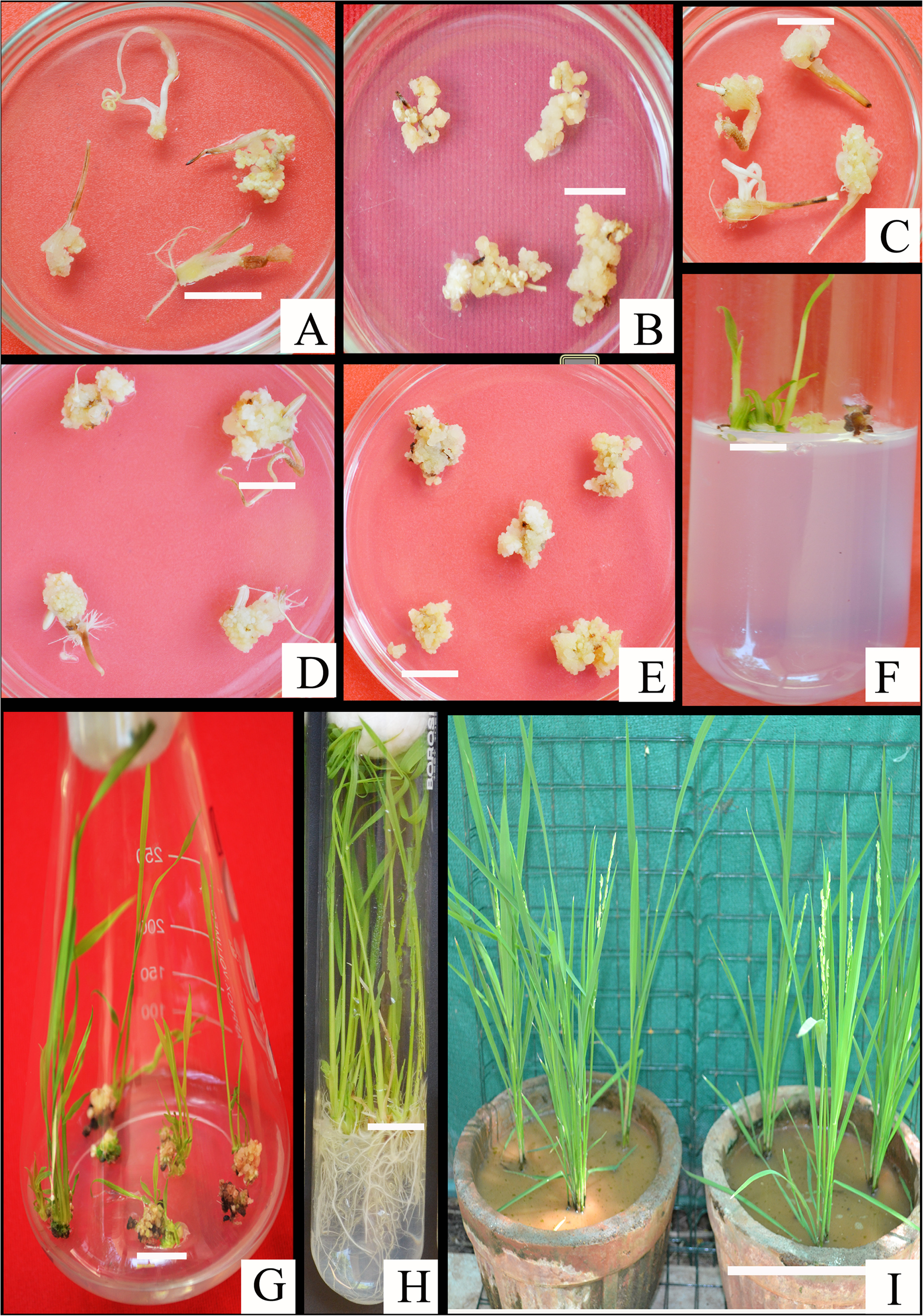

Supplement: Supplementary file 3 — Authors’ original file for figure 1 [file 40529_2013_97_MOESM3_ESM.tif]

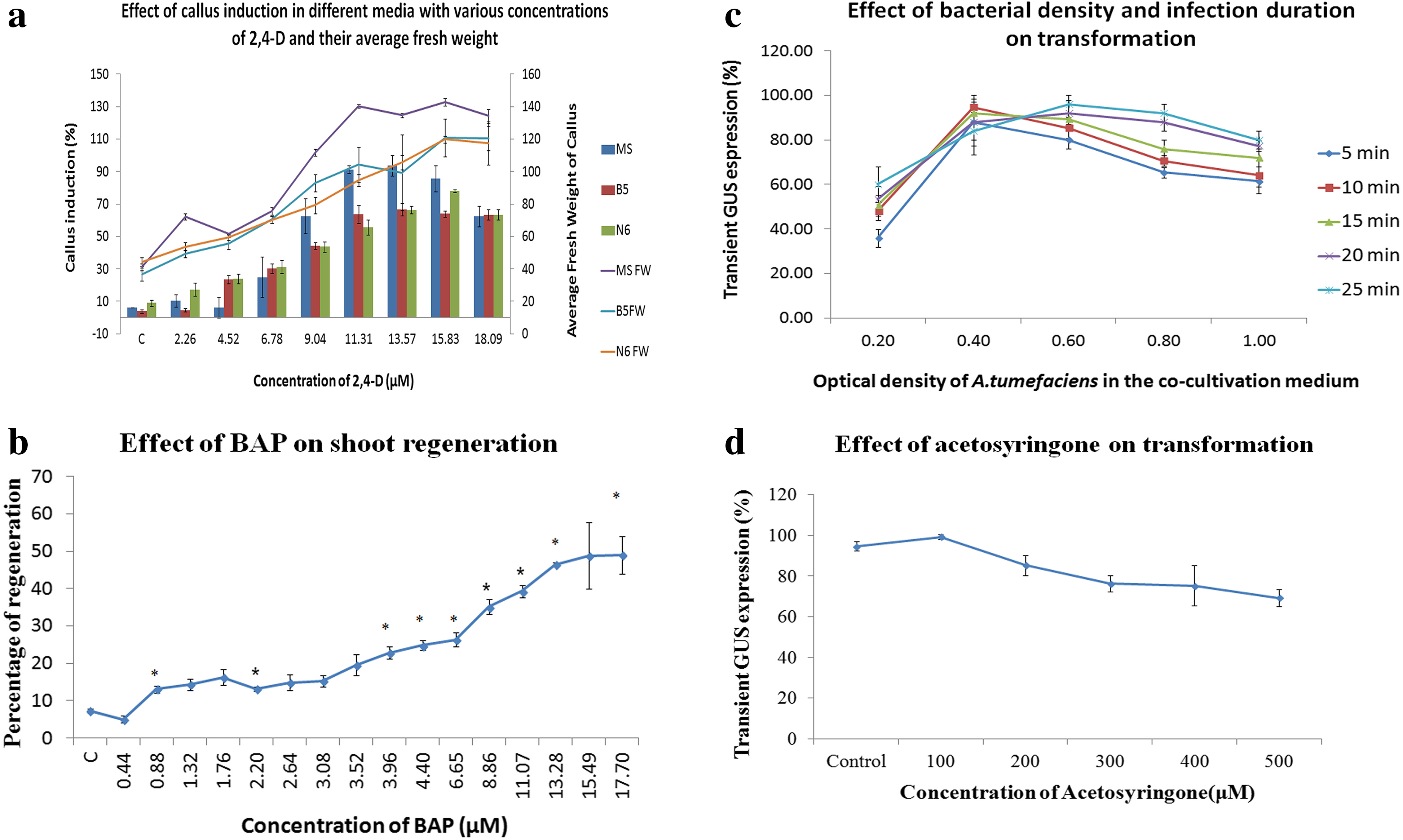

Supplement: Supplementary file 4 — Authors’ original file for figure 2 [file 40529_2013_97_MOESM4_ESM.tiff]

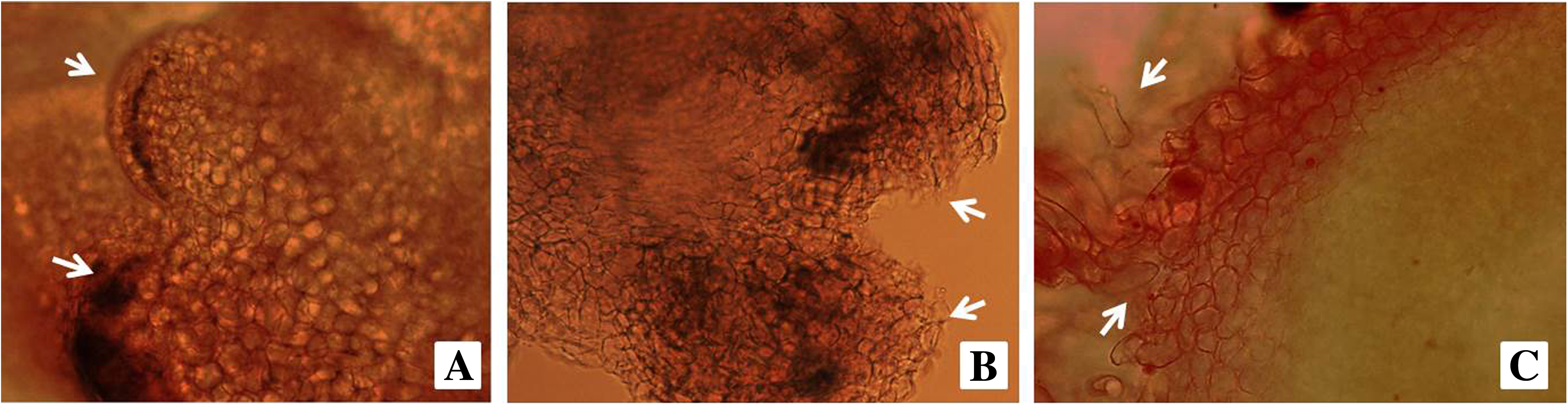

Supplement: Supplementary file 5 — Authors’ original file for figure 3 [file 40529_2013_97_MOESM5_ESM.tif]

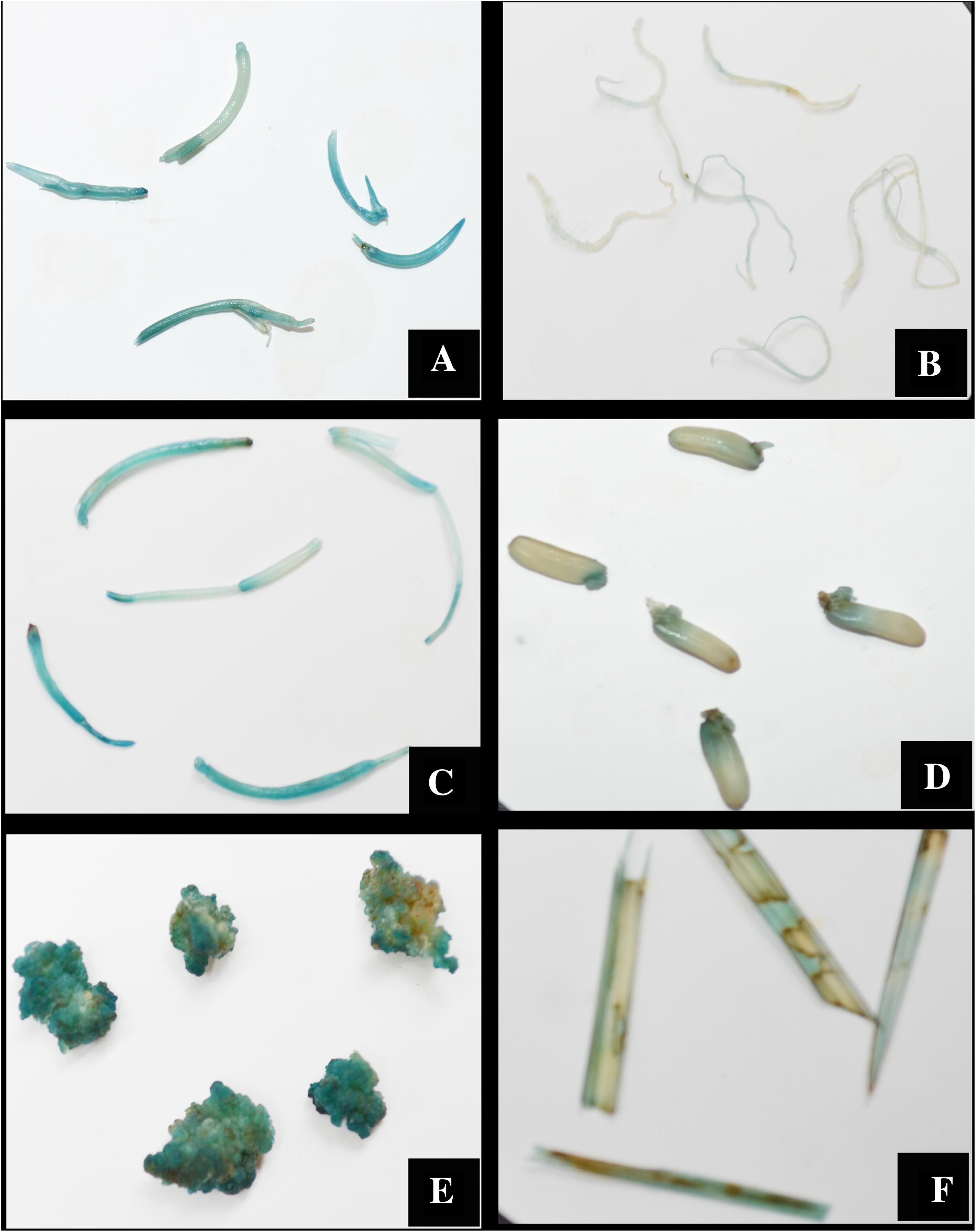

Supplement: Supplementary file 6 — Authors’ original file for figure 4 [file 40529_2013_97_MOESM6_ESM.tif]

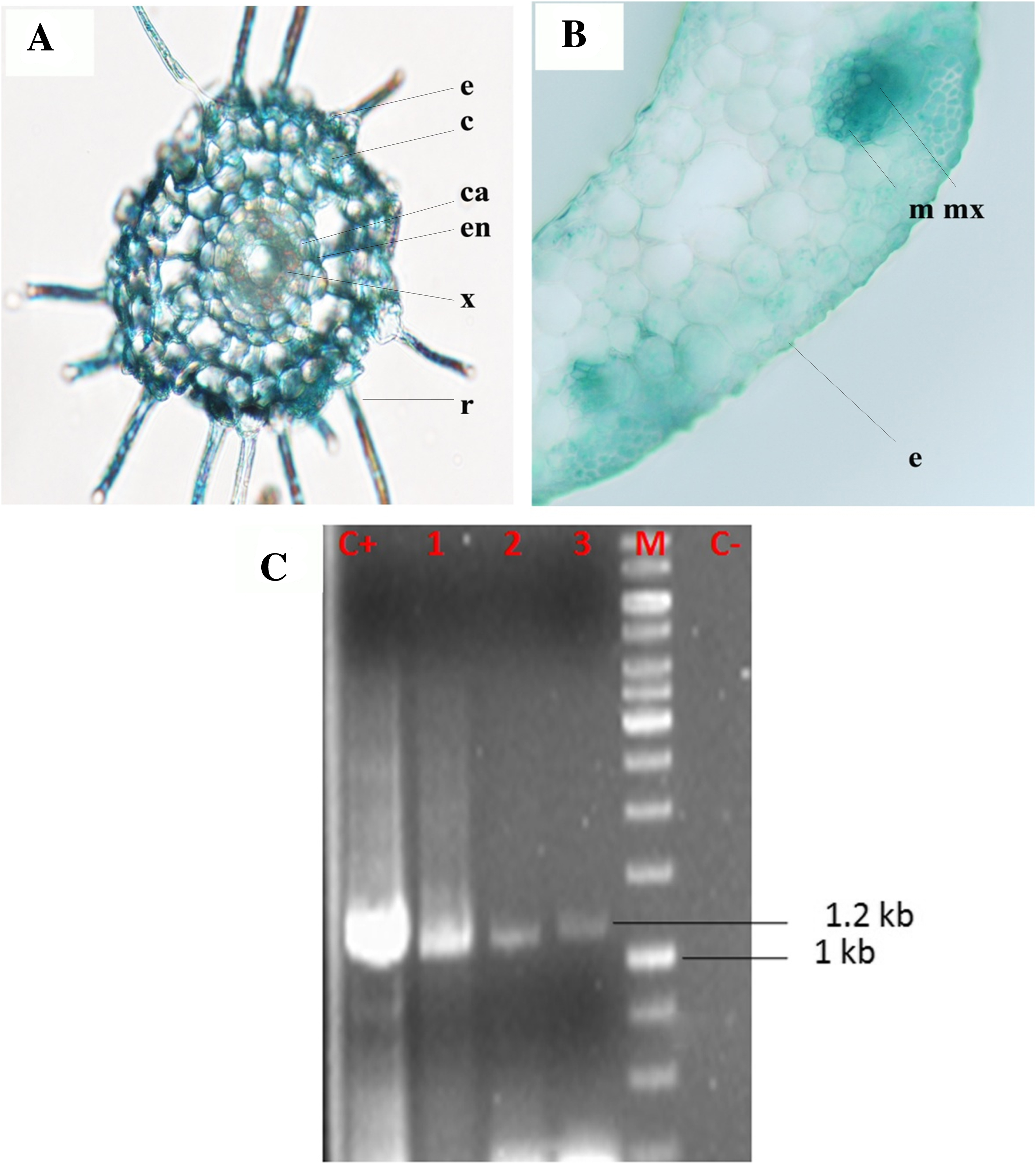

Supplement: Supplementary file 7 — Authors’ original file for figure 5 [file 40529_2013_97_MOESM7_ESM.tif]

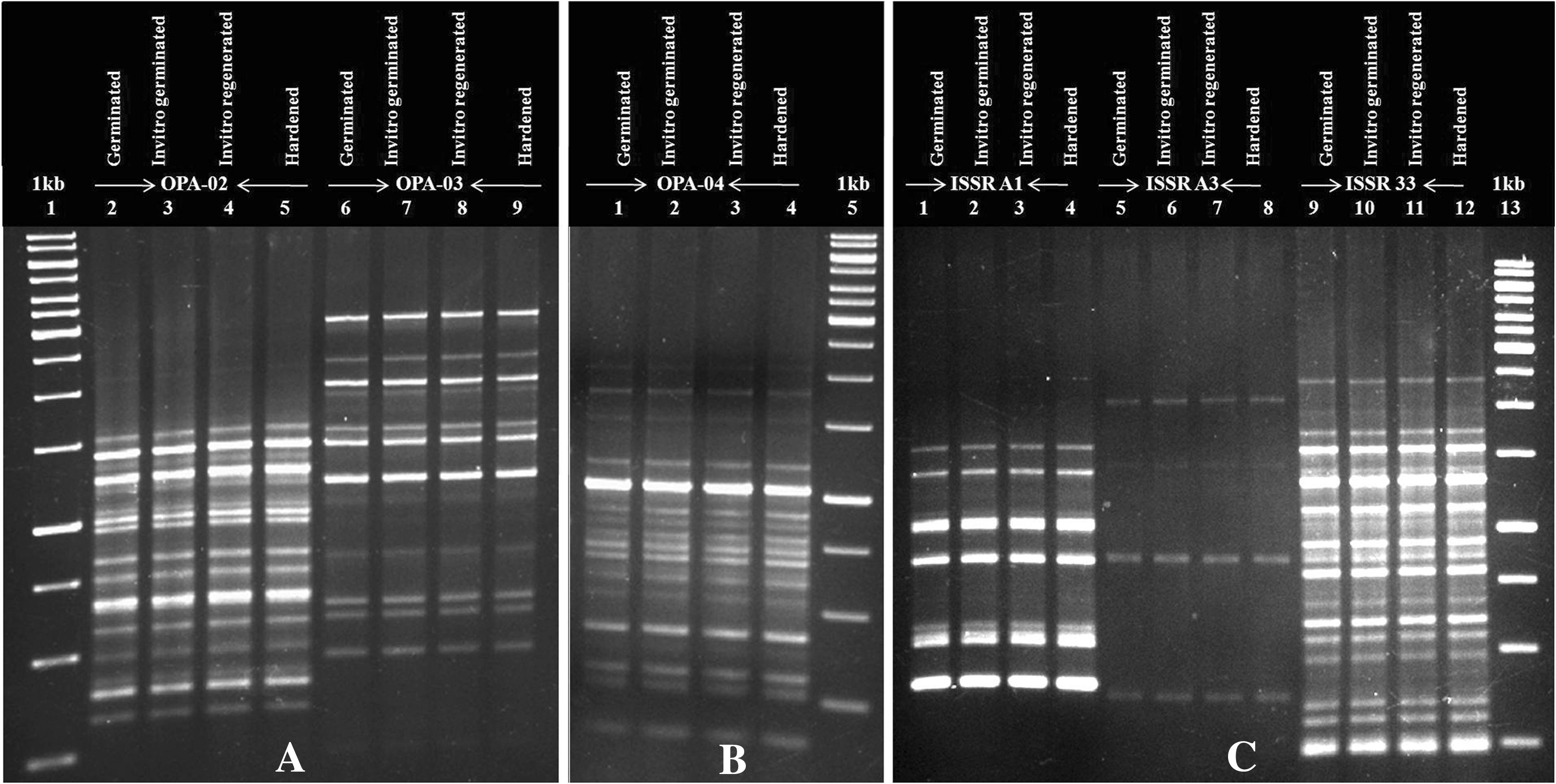

Supplement: Supplementary file 8 — Authors’ original file for figure 6 [file 40529_2013_97_MOESM8_ESM.tif]
